# AOJNMB in its second year of publication

**Published:** 2014

**Authors:** S. Rasoul Zakavi

**Affiliations:** Editor-in-chief

Asia Oceania Journal of Nuclear Medicine and Biology (AOJNMB) is now entering its second year of publication. After the publication of the first issue, we received a great deal of positive feedback from all around the world. The feedback ensured us that AOJNMB publication met a crucial need of the nuclear medicine community in the region and encouraged us to work harder.

AOJNMB introduction to a large number of readers was one of the main objectives in the first year of publication. Our enthusiastic editorial board and associate editors introduced this journal to their respective scientific societies. Also, free printed copies of AOJNMB were distributed in the annual meetings of European Association of Nuclear Medicine (EANM) in Lyon, World Association of Radiopharmaceutical & Molecular Therapy (WARMTH) in Manila, and Asian Regional Cooperative Council for Nuclear Medicine (ARCCNM) in Mumbai. This policy will be followed for introducing AOJNMB to a large number of nuclear medicine physicians and scientists. Our editorial board could be of significant help for further introduction of AOJNMB.

Visibility and wide availability significantly contribute to the success of a journal. There are many indexing and abstracting services which greatly increase the visibility of the articles and direct the researchers to relevant articles in the field. These services facilitate broader dissemination of the journal among scientists. Timely publication and frequent citation of the published articles in other journals are two main criteria that help indexing a journal in main scientific databases. Indexing in these databases is mandatory for bibliometric analyses which have been used for the assessment of the importance of a journal in the scientific world. Impact factor, SCImago journal rank factor, and Eigenfactor score are among the most common indices used for this purpose (1).

Although these factors have limitations and should be used with caution, they are the current available tools for journal ranking (2). AOJNMB is now indexed in DOAJ, EBSCO, IMEMR, Index Copernicus, ISC, Magiran, and some other local indexing databases. We will apply for citation in other major scientific databases as soon as the required number of articles has been published.

In the first year of publication, AOJNMB received various manuscripts mainly from Japan, Iran, and Korea; however, scientists from 9 different countries published their works in this volume. The average time from submission of original articles to first decision was 26 days, which demonstrates the commitment of our reviewers to the timely reviewing of the articles. The rejection rate for the original articles was 20% in the first year, which is acceptable for a newly published journal.

We sadly lost one of our valued editorial board members (Prof. Ajit Padhy) in the previous year. In the same period, five interested scientists from five different countries joined us in the editorial team. I would like to thank the editorial board, associate editors, and our reviewers for their invaluable comments, which play a key role in the success of our journal. Success depends on constancy of efforts and critical appraisal. We will keep the first and wish to receive your feedback for the second.
